# Supplementary material for: Splice-Junction-Based Mapping of Alternative Isoforms in the Human Proteome
Source: Cell Rep. Author manuscript; Available in PMC 2020 Jan 15. (PMC6961840; doi:10.1016/j.celrep.2019.11.026)
Supplement: 2 [file NIHMS1546469-supplement-2.zip › DF1/PXD010154/Liver/Liver_9_FGG_QVRPEHPAETEYDSLYPEDDI.pdf]

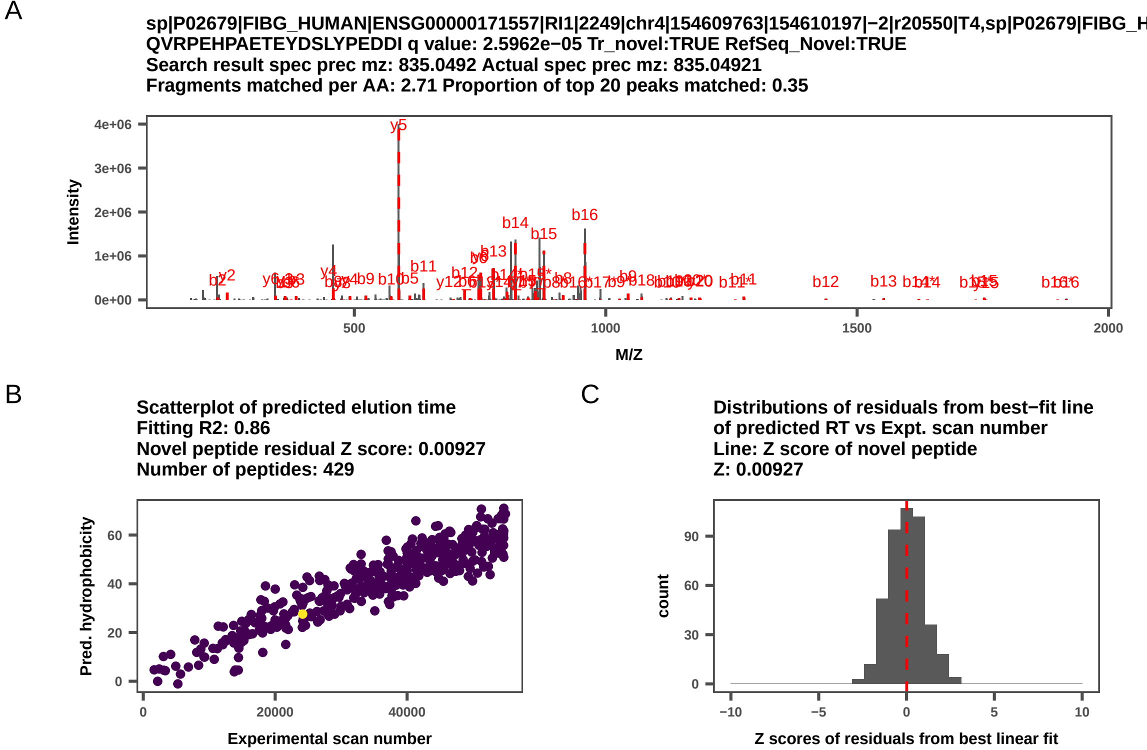
A
sp|P02679|FIBG_HUMAN|ENSG00000171557|RI1|2249|chr4|154609763|154610197|−2|r20550|T4,sp|P02679|FIBG_H
QVRPEHPAETEYDSLYPEDDI q value: 2.5962e−05 Tr_novel:TRUE RefSeq_Novel:TRUE
Search result spec prec mz: 835.0492 Actual spec prec mz: 835.04921
Fragments matched per AA: 2.71 Proportion of top 20 peaks matched: 0.35
Intensity
4e+06
3e+06
2e+06
1e+06
0e+00
y5
b14
b15
b16
b13
b11
b12
b10
b5
y4
y2
b9
b12
b13
b14*
b16*
M/Z
500
1000
1500
2000
B
Scatterplot of predicted elution time
Fitting R2: 0.86
Novel peptide residual Z score: 0.00927
Number of peptides: 429
Pred. hydrophobicity
60
40
20
0
0
20000
40000
Experimental scan number
C
Distributions of residuals from best−fit line
of predicted RT vs Expt. scan number
Line: Z score of novel peptide
Z: 0.00927
count
90
60
30
0
−10
−5
0
5
10
Z scores of residuals from best linear fit
